# Prevalence of human herpesvirus 8 infection in systemic lupus erythematosus

**DOI:** 10.1186/1743-422X-8-210

**Published:** 2011-05-09

**Authors:** Yu Sun, Shipeng Sun, Wenli Li, Bo Li, Jinming Li

**Affiliations:** 1 National Center for Clinical Laboratories, Beijing Hospital, Beijing, People's Republic of China; 2 Graduate School, Peking Union Medical College, Chinese Academy of Medical Sciences, Beijing, People's Republic of China; 3 Undergraduate School, College of Liberal Arts and Sciences, University of Colorado Denver, Denver, Colorado, USA

## Abstract

**Background:**

For decades, scientists have tried to understand the environmental factors involved in the development of systemic lupus erythematosus (SLE), in which viral infections was included. Previous studies have identified Epstein-Barr virus (EBV) to incite SLE. Human herpesvirus 8 (HHV-8), another member of the gammaherpesvirus family, shares a lot in common with EBV. The characteristics of HHV-8 make it a well-suited candidate to trigger SLE.

**Results:**

In the present study, serum samples from patients (n = 108) with diagnosed SLE and matched controls (n = 122) were collected, and the prevalence of HHV-8 was compared by a virus-specific nested PCR and a whole virus enzyme-linked immunoassay (EIA). There was significant difference in the prevalence of HHV-8 DNA between SLE patients and healthy controls (11 of 107 vs 1 of 122, *p *= 0.001); significant difference was also found in the detection of HHV-8 antibodies (19 of 107 vs 2 of 122, *p *< 0.001).

We also detected the antibodies to Epstein-Barr virus viral capsid antigen (EBV-VCA) and Epstein-Barr nuclear antigen-1 (EBNA-1). Both patients and controls showed high seroprevalence with no significant difference (106 of 107 vs 119 of 122, *p *= 0.625).

**Conclusion:**

Our finding indicated that there might be an association between HHV-8 and the development of SLE.

## Background

Systemic lupus erythematosus (SLE) is a disorder that affects multiple organ systems. Patients with SLE display abnormalities in immune system functions. For instance, it causes enhanced B cell function and the production of numerous autoantibodies. The etiology of SLE has not been completely elucidated now, however, there is striking evidence indicating that both genetic and environmental factors contribute to the development of SLE [1]. In other words, genetic predispositions play its role under the stimulation of environmental agents.

A number of viruses, including parvovirus B19 [2], Epstein-Barr virus (EBV) [3,4], human papillomavirus (HPV) [5,6], cytomegalovirus (CMV) [7], and some endogenous retrovirus [8], have been implicated as possible environmental factors contributing to the onset and/or exacerbation of SLE. One cogent example is EBV infection, which has been identified as the environmental risk factor most closely associated with SLE [9].

Human herpesvirus 8 (HHV-8), or Kaposi's sarcoma (KS)-associated herpesvirus, have a well-established role in the pathogenesis of KS. It belongs to the gammaherpesvirus subfamily and is closely related to Epstein-Barr virus [10]. HHV-8 and EBV have many similarities: Both of them are able to infect and build up latency in B lymphocytes. At the same time, they all have the abilities of producing a number of proteins responsible for maintaining viral episomes, promoting B-cell survival and proliferation, inhibiting p53 and Rb, and most interesting of all, encoding a large number of cellular homologs. Two results can be reached due to the production of cellular homologs. One is structural mimicry, as Epstein-Barr virus nuclear antigen-1 (EBNA-1) mimics human autoantigens Sm and Ro, which has been regarded as one of the possible mechanisms contributing to the onset of autoimmunity [1]; another one is functional mimicry, as HHV-8 expresses the homologs of Bcl-2 to protect virus-infected cells from apoptosis, by which the viral proteins are able to substitute their cellular counterparts to act [11].

Since the characteristics of HHV-8 itself and the correlation between HHV-8 and EBV, we hypothesized that the infection of HHV-8 was associated with SLE. From patients with diagnosed SLE and healthy controls, serum samples were collected to compare the prevalence of HHV-8 in order to determine this association. Due to the pathogenetic role of EBV in the development of SLE, serologic tests were also performed to determine the occurrence of it.

## Methods

### Study populations

All studies were carried out with the approval of the local hospital ethical committee, patients (n = 108) with SLE who fulfilled the 1997 revised American College of Rheumatology criteria for SLE [12] and healthy controls (n = 122) were enrolled in the study. The mean age and female to male ratio were matched between patients with SLE and healthy controls. (See Table 1 for demographics) All the participants enrolled in the study were ethnic Chinese. Serum samples were obtained from clinics at Xuanwu hospital, Beijing, China, between 2009 and 2010. In addition, those samples were stored at -80°C within one hour of collection until tested. Informed consent was obtained from each subject.

**Table 1:** Demographic data for study groups

	Groups		
		
Characteristics	SLE Patients	Normal Donors	*P *value
Demographic Data			
No. of participants	107	122	
age (mean ± SD, yrs)	34.1 ± 12.1	33.5 ± 7.6	.72
Sex			
Female (%)	93 (86.9)	111 (91.0)	.33
Male (%)	14 (13.1)	11 (9.0)	

### Virus-specific PCR detection

DNA was extracted from 0.5 ml of each serum sample using the QIAamp Viral RNA Mini Kit (Qiagen), following the instruction of the vendor. A fragment of β-actin (304 bp) was amplified to confirm successful extraction of DNA and no PCR inhibitors were present. One patient was excluded in this study because of the failure to amplify the fragment of β-actin.

**Figure 1:**
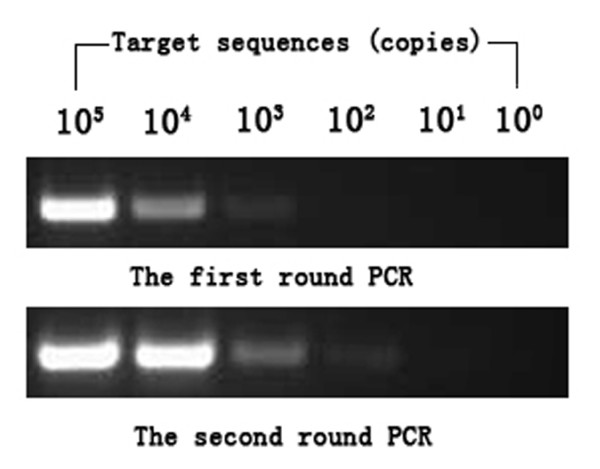
**Nested PCR sensitivity**. Positive control was serially diluted to be amplified by nested PCR. The detection limit of the nested PCR assay used in this study was 100 copies. For the first round PCR, > 10^3 ^target sequences could be detected.

DNA extracted from samples was examined for the presence of a 233-bp-long fragment for HHV-8 (ORF26) using a nested PCR protocol as previously described [13]. The sensitivity of the nested PCR was determined by series dilutions of the positive control, which has a detection limit of 100 copies. For the first round PCR, > 10^3 ^target sequences could be detected (Figure 1). Each first-round reaction was performed in a 25 μl volume containing, 1.5 mM Mg^2+^, 200 mM each dNTP, 0.4 μM each primer and 2 × Green Gotaq reaction buffer (Promega) to which 100 ng DNA extract or control was added. Second round reaction mixes were identical to the first round and the templates were 1 μl products from the first round reactions. Reaction conditions were one cycle of 95°C for 2 min, thirty-five PCR cycles consisting of denaturation at 95°C for 30 s, annealing at 50°C for 30 s and extension at 72°C for 30 s were run per round with a 7 min final extension at 72°C for both first round and nested PCR. PCR reactions containing all PCR reagents without DNA template served as negative control. The 233-bp-long fragment for HHV-8 ORF26 was amplified by means of a PCR-driven overlap extension protocol and subsequently cloned into the pGEM-T vector system kit (Promega) to serve as positive control. The first and nested PCR products were analyzed in 1.5% TAE agarose gels stained with ethidium bromide. For positive samples, the nested PCR products was sequenced.

To minimize possible contaminations, Serum samples from patients and healthy controls were randomly selected and divided into several groups to extract DNA; with each group contained sixteen samples, three separate reactions of negative controls and one positive control were analyzed by PCR. DNA extraction, PCR and gel electrophoresis were performed in separate rooms. Positive samples were then validated by repeat testing.

### Serological assays

To detect antibodies to HHV-8, serologic testing was performed by a commercial ELISA (ABI) based on whole virus lysate, according to the manufacturer's instructions. This assay has a sensitivity of 77% and a specificity of 99% [14].

The occurrence of infection with EBV was determined by measuring IgG antibodies against EBV-VCA and EBNA-1 (EUROIMMUN), following the manufacturer's instructions.

### Statistical analysis

Data were expressed as the mean ± SD for normally distributed variables. Statistical analysis was performed with SPSS v. 16.0. The association between HHV-8 infection and the occurrence of SLE was assessed using the chi-square test; the association between EBV infection and the occurrence of SLE was assessed using the Fisher's Exact Test. The association Data with a *P *value of less than 0.05 were considered significant.

## Results

### Detection of HHV-8 DNA

**Figure 2:**
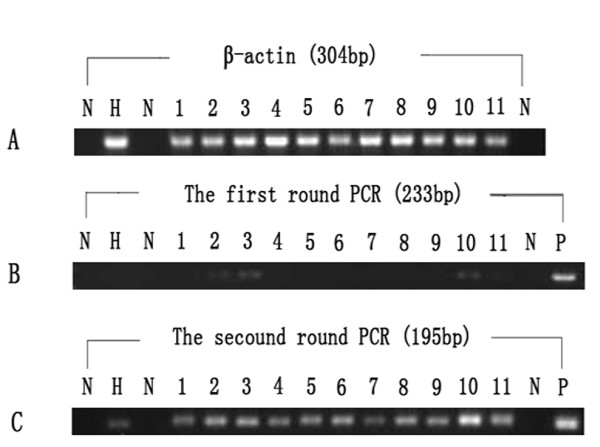
**Positive samples for HHV8**. A: Amplification of β-actin (304 bp), B: the first round PCR (233 bp), C: the second round PCR (195 bp). Lane N: negative control, Lane H: positive sample of healthy controls, Lane 1-11: positive samples of patients with SLE, Lane P: positive control.

DNA isolated from sera was used as template to screen for target sequence. Of the 107 patients with SLE, 11 (10.3%) were determined positive for HHV-8 ORF26; whereas in healthy controls, 1/122 (0.8%) was positive. The results between those two groups was statistically different (*p *= 0.001, by χ^2 ^test). All the repeat testing for positive samples showed consistent results (Figure 2). DNA sequence assay for positive PCR products showed expected results for HHV-8 ORF26 sequences.

### Serologic finding

Sera showed positive for IgG antibodies either against EBV-VCA or against EBNA-1 were considered to be EBV-positive. Both lupus patients and healthy controls exhibited high incidence of infection with EBV (patients: 106/107 [99.1%]; controls: 119/122 [97.5%]), with no significant difference (*p *= 0.625, by Fisher's Exact Test) was found.

Nineteen (17.8%) of 107 lupus patients and 2 (1.6%) of 122 matched controls were proven to be seropositive, respectively. The results between those two groups was statistically different (*p *< 0.001, by χ^2 ^test). As shown in table 2, of 19 those lupus patients showed seroreactivity, ten were positive for both HHV-8 sequences and antibodies; whereas one heathy control showed positive for both HHV-8 sequences and antibodies of the two healthy controls showed seroreactivity. All of the participents who were positive for HHV-8 DNA and HHV-8 antibodies showed positive for EBV antibodies.

**Table 2:** Detection of HHV-8 DNA and antibodies

	Groups			
	
HHV-8 antibodies	Lupus patients, No.		Normal controls, No.	
	
	DNA sequences (+)	DNA sequences (-)	DNA sequences (+)	DNA sequences (-)
Positive	10	9	1	1

Negative	1	87	0	120

## Discussion

The results from PCR assays and serologic testing in our study showed HHV-8 infection was more prevalent in patients with SLE than in healthy controls. We found a relatively low prevalence of HHV-8 in healthy controls, which was similar with other studies on the epidemiology of HHV-8 infection [15,16], suggesting that the prevalence of HHV-8 infection in China is low. Recently, a survey conducted in the northwest of China revealed that 20.4% of blood donors were seropositive for HHV-8 [17]. This observation suggested that the prevalence of HHV-8 infection varies depending on different regions.

It is noteworthy to point out that the nested PCR is a very sensitive tool with low detection limit, and serum has been used to detect HHV-8 DNA [18-20]. However, low detection rate of PCR detection has been observed compared to that of serologic assays, which dues to the low copy number and intermitent presence of HHV-8 DNA in serum [21-25].

An important finding of this study is the increased prevalence of HHV-8 in patients with SLE, which indicated that there might be an association between the development of SLE and HHV-8. One acceptable explanation may be the susceptibility of viral infection in patients with SLE. As a disorder with impairment of the immune system, SLE may lead to abnormalities of humoral and cell mediated immune responses, cytokine milieu and complement system. Furthermore, due to the application of immunosuppressant agents in routine treatment, patients with SLE may be predisposed to HHV-8 infection, thus resulting in a higher detection rate of HHV-8 infection. This predisposition has been observed in some transplant recipients and patients with autoimmune disease or malignancy who have received high dose of immunosuppressant agents [10], which leads to iatrogenic KS.

On the other hand, relative to passive infection, is there any possibility that HHV-8 actively involve in the development of SLE? The pathogenic role of EBV in SLE has been confirmed. As the gammaherpevirus homolog for HHV-8, the virus can infect B cell latently, providing the latent viral protein Epstein-Barr virus nuclear antigen-1 (EBNA-1). EBNA-1 may molecularly mimic the common human antigenic targets for the lupus autoantigens. To date, only Sm and Ro have been identified to involve in molecular mimicry between EBV infection and SLE autoimmunity, which account for about 40% of all SLE [9,26]. In our study, we screened the seroprevalence of EBV among the study subjects. High frequencies of EBV infection were observed and no difference was found between two groups. This finding was consistent with the fact that EBV is a ubiquitous virus and indicated that the infection of EBV is highly prevalent in China. Also, we suspected that EBV might infect individuals long before the onset of SLE, rather than secondary infection resulting from impaired immune system or the application of immunosuppressants.

Several mechanisms have been proposed by which viruses trigger autoimmune diseases, including adjuvant effects, molecular mimicry, bystander activation and epitope spreading [1]. In these mechanisms: (a) viruses can act as adjuvants to strengthen the function of antigen-presenting cells (APCs) by pattern-recognition receptors (PRRs), which recognize a large number of molecular patterns present in viruses, resulting in the production of inflammatory factors and in turn to tissue damage; (b) viral antigens cross-reactive with self antigens allow the activation of autoreactive T cells through molecular mimicry; (c) self-antigens released from damaged tissue were presented by activated APCs to autoreactive T cells in the process of bystander activation; (d) additional autoreactive T cells responded for different portion of the same protein or a different protein is stimulated via epitope spreading. Considering the characteristic of clinical and immunological heterogeneity, and the large pool of autoantigens in patients with SLE, it is possible that additional epitopes of different proteins have the potential of molecular mimicry, which means existing achievements are far from fully investigated. Previous investigations have identified that HHV-8 encodes a large number of cellular homologs. For example, HHV-8 encodes viral interleukin-6 (vIL-6) in B cells [27,28], intriguingly, resulting in B-cell survival and proliferation. Therefore, HHV-8 could be regarded as a superior candidate due to constant immune stimuli triggered by the lifelong latency of itself.

## Conclusions

In a relatively large population of Chinese patients with SLE, we demonstrated that the prevalence of HHV-8 was increased than that of in healthy controls. Our results therefore implicate that there might be an association between HHV-8 and the development of SLE. This observation raises a series of important questions: Is HHV-8 infection a critical factor in the pathogenesis of SLE or a passenger virus? Whether HHV-8 can trigger and/or exacerbate SLE as its gammaherpesvirus homologous EBV does? Is there other viruses play pathogenic roles in the development of SLE? Considerable more investigations, hopefully, will be done in this area.

## Competing interests

The authors declare that they have no competing interests.

## Authors' contributions

YS conducted the experiments, performed the statistical analysis and drafted the manuscript. SS & WL participated in the design of the study and collected specimens and data from the study population. BL modified the manuscript. JL generated the concept for the study, participated in its design and coordination. All authors read and approved the final manuscript.
